# Home and Workplace Neighborhood Socioeconomic Status and Behavior-related Health: A Within-individual Analysis

**DOI:** 10.1093/abm/kaaa116

**Published:** 2021-02-13

**Authors:** Auriba Raza, Martin Claeson, Linda Magnusson Hanson, Hugo Westerlund, Marianna Virtanen, Jaana I Halonen

**Affiliations:** 1 Stress Research Institute, Department of Psychology, Stockholm University, Stockholm, Sweden; 2 Department of Psychology, University of Eastern Finland, Joensuu, Finland; 3 Department of Health Security, National Institute for Health and Welfare, Helsinki, Finland

**Keywords:** Longitudinal study, Home neighborhood, Work neighborhood, Health-related behaviors, Socioeconomic status

## Abstract

**Background:**

The influence of individual and home neighborhood socioeconomic status (SES) on health-related behaviors have been widely studied, but the majority of these studies have neglected the possible impact of the workplace neighborhood SES.

**Objective:**

To examine within-individual associations between home and work place neighborhood SES and health-related behaviors in employed individuals.

**Methods:**

We used participants from the Swedish Longitudinal Occupational Survey of Health who responded to a minimum of two surveys between 2012 and 2018. Data included 12,932 individuals with a total of 35,332 observations. We used fixed-effects analysis with conditional logistic regression to examine within-individual associations of home, workplace, as well as time-weighted home and workplace neighborhood SES index, with self-reported obesity, physical activity, smoking, excessive alcohol consumption, sedentary lifestyle, and disturbed sleep.

**Results:**

After adjustment for covariates, participants were more likely to engage in risky alcohol consumption when they worked in a workplace that was located in the highest SES area compared to time when they worked in a workplace that was located in the lowest SES area (adjusted odds ratios 1.98; 95% confidence interval: 1.12 to 3.49). There was an indication of an increased risk of obesity when individuals worked in the highest compared to the time when they worked in the lowest neighborhood SES area (1.71; 1.02–2.87). No associations were observed for the other outcomes.

**Conclusion:**

These within-individual comparisons suggest that workplace neighborhood SES might have a role in health-related behaviors, particularly alcohol consumption.

## Teaser

Comparing an individual to his/herself at two time points suggested that workplace neighborhood SES has a role in health-related behaviors, particularly in risky alcohol consumption

## Introduction

Individuals’ behaviors such as physical inactivity, sedentary lifestyle, smoking, alcohol consumption, and high body mass index (BMI) are important determinants of health and have been associated with an increased risk of morbidity and cardiovascular as well as all-cause mortality [1–3]. As these health-related behaviors are modifiable, they are crucial in preventing chronic diseases [4]. While strong individual-level socioeconomic gradients exist in these health-related behaviors [5–8], they may also be influenced by environmental characteristics of neighborhoods where people live, work, or spend their leisure time. In neighborhood-level studies, for instance, living in a low socioeconomic status (SES) neighborhood has been associated with binge drinking, physical inactivity, obesity, and smoking [5, 9–12].

Hypothesized mechanisms underlying these associations include environmental and contextual factors which capture how communities and neighborhoods affect individuals’ health and health-related behaviors [13, 14]. Neighborhood socioeconomics, for instance education, unemployment, poverty, and homeownership are major contextual factors that might drive both social factors and built characteristics of a neighborhood [14]. Studies have reported that neighborhoods with low SES might have low social cohesion, low social control, and high crime rates [15, 16]. Area-level social capital has been associated with physical activity [17], high crime rates to increased smoking prevalence [18] and increased BMI through decreased physical activity and decreased perceived safety [19]. Individuals with high or medium SES were reported to have a greater probability of good sleep quality compared to individuals with a low SES [20]. Anxiety, depression, and health status are important additional determinants, which are more prevalent in lower SES were observed to be associated with poorer sleep quality [20]. In addition, high densities of alcohol and smoking outlets and fast-food facilities, low esthetic qualities, and absence of adequate facilities for physical activity [21, 22] might lead to poor health-related behaviors of individuals living in low SES neighborhoods. While the mechanisms between associations of workplace neighborhood SES and health are likely similar to home neighborhoods, a majority of the research has been around home neighborhood SES [5, 9–12, 23], and less is known about impact of additional contextual environments such as those around workplaces, on behavior-related health [24–26].

People spend a considerable amount of waking time in their workplace. Workplace neighborhoods may thus play a role in shaping health-related behaviors. Few studies have reported findings of associations between workplace neighborhood and health [24, 27]. One linked healthy food availability around workplace environments with lower BMI [24] and another reported a higher density of physical activity facilities in workplace neighborhoods than in home neighborhoods [27]. Furthermore, the potential synergistic effect of home and work neighborhood SES on health-related behaviors has not been explored in a longitudinal setting [24].

The majority of studies on neighborhood health-related behaviors, especially the few examining workplace neighborhoods, have been cross-sectional [10, 12, 23–28], limiting the ability to draw a causal inference as the chronological order of exposure and outcome cannot be determined. These methodological limitations can be mitigated by a longitudinal study design with repeated measurements [29]. Within-individual analyses can assess whether individuals had healthier behaviors when they were living or working in a higher SES neighborhood compared to another time when the same individuals were living or working in lower SES neighborhoods [29, 30]. The within-individual design adjusts for unmeasured time-invariant characteristics of the individuals [29], such as personality, as individuals are compared with themselves when living or working in neighborhoods with different SES. Thus, a within-individual estimation can provide insight into potential causal processes linking neighborhood SES and health-related behaviors [9, 29].

In this study, we used repeated measures from a population-based survey to examine longitudinal associations of home and workplace neighborhood SES separately and jointly with health-related behaviors in gainfully employed individuals. We hypothesize that individuals when living or working in a higher SES neighborhood would have a lower likelihood of poor health-related behaviors compared to times they were living or working in lower SES neighborhoods.

## Materials and Methods

### Study Population

We used data from Swedish Longitudinal Occupational Survey of Health (SLOSH), a national longitudinal survey of work-life participation, work environment, and health and wellbeing that started in 2006 [31]. SLOSH participants consist of the respondents of the Swedish Work Environment Surveys 2003–2011 who were initially sampled biennially from 2003 to 2011 from the Labor Force Surveys by Statistics Sweden [32]. The SLOSH cohort participants, 16–64 years of age at the time of first response, are followed-up biennially and are sent two versions of the questionnaire: (i) gainfully employed for at least 30% of full-time or (ii) employed less than 30% or unemployed. Seven biennial waves of SLOSH from 2006 to 2018 have been conducted.

One of the objectives of this study is to determine the association of work neighborhood SES on behavior-related health. Therefore, we only included participants who were gainfully employed for at least 30% of full-time at the time of the survey. Further inclusion criterion was a response to outcome-related questions in at least two of the four SLOSH waves between 2012 and 2018. We excluded participants with missing information on covariates or objectively measured exposure. Total number of gainfully employed individuals who responded to any SLOSH wave between 2012 and 2018 was 14,618. We had an analytical sample of 12,932 participants after applying our exclusion criteria. This analytical sample contains individuals eligible for the analyses. Among the included there were slightly more women (58% vs. 52%) and those with children (28% vs. 18%) than among excluded, and less those with chronic diseases (41% vs. 49%).

### Home and Work Neighborhood Socioeconomic Status

Our neighborhood units consist of areas within a Euclidean distance of a 500 or 1,000 m radius around the home and workplace address. Address information was from the end of December on the preceding year of the included surveys that were conducted in the spring of 2012, 2014, 2016, and 2018. Concept of SES is multidimensional and single variables do not capture the complexity of the concept of SES (whether at an individual or group level) [33]. Therefore, we used a summary neighborhood SES score as the main indicator of the SES of the neighborhood. To create the summary score, we used three variables representing the dimensions of income and social status; mean household income, low education (percentage of adults above 18 years with only elementary school education), and unemployment (percentage of unemployment) within 500 and 1,000 m radius of home or workplace address. These variables have been predominantly used to characterize neighborhood SES in the previous literature [11, 23, 24, 34–36]. These three socioeconomic determinants were obtained from Statistics Sweden. Since SLOSH surveys were conducted in spring, the data for above-mentioned three variables were obtained for the years preceding the SLOSH surveys, that is, 2011, 2013, and 2015 for the SLOSH waves 2012, 2014, and 2016, respectively. For the 2018 SLOSH wave, the reference year was 2016 as data were not available for 2017.

Prior to calculation of summary scores for neighborhood SES, we log-transformed mean income and educational attainment for a more normalized distribution. Low education and unemployment rates were coded as additive inverse to get the lowest values for the highest SES. For each SES determinant, standard *z*-scores (mean = 0, *SD* = 1) were derived. We calculated neighborhood SES by taking the mean values across the three *z*-scores. Using the mean values neighborhood SES was divided into quartiles, the highest quartile indicating the highest neighborhood SES. Our neighborhood SES measure was operationalized as quartiles as the majority of the neighborhood and behavior-related health research used quartiles and thus this facilitates comparison with the previous research [5, 9–11, 21, 23, 26]. Furthermore, our research question compares individuals moving from low to high neighborhood SES thus use of categorized measure was reasonable.

Summary measures for each participant’s “home and work” neighborhood SES were created using a previously applied approach [24]. We had information on the number of hours participants spent at work and commuting to and from work per week and used this information to estimate the number of hours spent at home per week. We calculated time-weighted averages of the home and work neighborhood SES. Weights were proportional to the number of hours spent at each location during the week.

### Behavior-related Health Outcomes

From survey responses, we included physical inactivity, sedentariness, obesity (based on BMI), smoking, problem drinking, and disturbed sleep as outcomes. All outcome variables were collapsed into dichotomous variables as not all of them could be used as continuous variables due to categorized nature of the responses. The choice of cut-offs was based either on the previous literature or decided by the authors after accessing the information provided by the respondents.

*Physical inactivity* of participants was evaluated by using a question “How much do you exercise? including walking and cycling to and from work” with response alternatives: never exercise, move very little or take occasional walks, exercise now and then, and exercise regularly. We categorized participants as physically inactive if they selected the first or second response alternative and active if they selected the third or the last alternative [37].

*Obesity* was based on BMI that was calculated as self-reported weight in kilograms (kg) divided by self-reported height in meters squared in each follow-up. Obesity was dichotomized as Obese (BMI ≥ 30) versus not obese (BMI < 30).

*Smoking status* of participants was collected using a question, *Do you smoke?* and categorized as smokers (daily or occasional smokers), and nonsmokers (never or former smokers).

*Risky alcohol consumption* was assessed using the modified Cut-Annoyed-Guilty-Eye (CAGE) [38] questionnaire. It includes the following questions for participants reporting any drinking: (i) Have you felt you should cut down on your drinking? (ii) Have people annoyed you by criticizing your drinking? (iii) Have you felt bad or guilty about your drinking? (iv) Have you had a drink first thing in the morning to steady your nerves or get rid of a hangover? Participants’ alcohol consumption was categorized as risky if they reported a minimum of two problem drinking behaviors [38].

*Disturbed sleep* was based on the Disturbed Sleep Index from the Karolinska Sleep Questionnaire [39]. Participants were asked if they had difficulties falling asleep, restless sleep, repeated nocturnal awakenings, and premature awakening. For each question, there were six response alternatives: never, rarely, few times per month, 1–2 times per week, 3–4 times per week, and 5 or more times per week. We defined disturbed sleep as having one or more sleep problems 3–4 times a week or more [37, 40].

*Sedentariness:* In the 2014, 2016, and 2018 waves, two questions were used to assess sedentary behavior: (i) “on average, how many hours do you spend sitting in a weekday; (a) during working day, (b) during travel time to and from work, and (c) during spare time.” For each part, there were five response alternatives: 0–1, 2–3, 4–5 h, 6–7, and 8 h or more and (ii) “How often do you usually take shorter breaks to move when sitting for longer hours? (a) at work, and (b) in spare time” with response alternatives; never/almost never, quite seldom, now and then, quite often, and frequently. We only included participants who responded to all sections of the above two questions. The mean of the hours spent sitting during the working day, commuting, and spare time were in each wave used as cutoffs for that wave. We formed a combination variable based on the two questions (sitting hours and taking breaks). Participants’ behavior was characterized as sedentary if they spent 7.5 (mean) hours or more sitting without taking breaks quite often and active if otherwise. Previous studies on sedentary behavior categorized participants’ behavior as sedentary based on reading of accelerometers or inclinometers or daily diaries separately or combined [41] but our choice of categorization was derived by the information on sitting time provided by study participants.

### Covariates

Self-reported sociodemographic variables were a number of children (one or more children under 12 years) and occupational position (low = manual employees, intermediate = nonmanual employees, high = professionals, and self-employed). This categorization was based on the Swedish socioeconomic classification [42] and study participants were categorized based on the information provided about occupation, detailed job title, and main tasks at work. Information on age and civil status (married/cohabiting vs. not) was obtained from registers.

Health and work-related variables included chronic diseases, symptoms of depression, and psychological job strain. For chronic diseases, if participants had hypertension or cardiovascular disease or diabetes or rheumatic disorders or musculoskeletal disorders during the past 2 years they were coded as having chronic disease [37].

In all waves, symptoms of depression were evaluated using a six-item subscale of the (Hopkins) Symptom Checklist (SCL) resulting in SCL-Core Depression scale [43, 44]. Respondents were asked to score on a five-category scale the extent that they felt blue, had no interests in things, were lethargic or low in energy, were worrying too much about things, blamed themselves for things, and felt everything is an effort. For each item, scores were summed to get a continuous scale assessing the severity of depression. Using the continuous scale, we created a binary variable with a cut-off score of ≥17 [44]. A score of 17 has been identified as the best cut-point for major depression (sensitivity 0.68, specificity 0.98) which predicted subsequent purchases of antidepressants as well as hospitalizations with a depressive episode [44].

Job strain is defined as high job demands and low job control and heavy drinkers are more likely to report job strain [45]. Questions assessing job strain were based on Karasek’s job demand-control model in all waves [46]. A Demand Control Questionnaire has five job demand items: (i) working fast, (ii) working hard/intensively, (iii) no excessive amount of work/too much effort, (iv) enough time, and (v) conflicting demands, while job control has six control items: (a) learn new things, (b) high level of skill, (c) creativity/initiative, (d) repetitive work, (e) a lot of say/what to do, and (f) little freedom/how to do. In each wave for each participant, we created a mean of all five job demand and six job control items and used a median of means as a cut-off point. Participants with mean demands scores above the median and mean control scores below the median were categorized as having job strain [47].

These covariates were chosen as they have been linked to neighborhood SES and behavior-related health [26, 34, 45, 46].

### Statistical Analyses

We applied a fixed-effects approach [48], also known as within-individual or case-crossover design, and conditional logistics regression to analyze associations between home- and work-neighborhood SES and health-related behaviors in gainfully employed individuals. We used three exposure measures, that is, home and work neighborhood SES in separate models and as a time-weighted (“home and work”) variable. The main exposure variable used in the analyses was neighborhood SES within 500 m radius of home and workplace address. Small neighborhood of 500 m, on average 6–10 min walking distance from the addresses might correlate well with how residents perceive their neighborhoods.

We included participants who responded to a minimum of two waves as the within-individual method requires information from participants who report unfavorable health behavior (“case” situation) in one wave and favorable health behavior (“control” situation) in another wave. Those whose neighborhood SES changed between the waves were included in each analysis as “informative.” Since each case serves as its own control, time-invariant individual characteristics are controlled by the design [48].

In the analyses, our first model (Model 1) included adjustments for age, occupational position, marital status, and presence of children as covariates. In subsequent models, we additionally adjusted for chronic disease and depressive symptoms (Model 2); and job strain (Model 3). All covariates were included as time-varying variables.

Sex differences have been reported in prevalence of sleep problems [49], alcohol consumption [50], and smoking [11]. Therefore, possible effect modification of the association between neighborhood SES and behavior-related health by sex was investigated by constructing multiplicative interaction terms.

As sensitivity analyses, for health-related behaviors demonstrating associations with the main exposure, we ran home neighborhood SES models adjusted for work neighborhood SES and vice versa. Data on socioeconomic determinants was based on income, education attainment, and employment status of individuals living in the neighborhoods. In 2010 in Sweden, about 12% of workplace areas with concentrations of workplaces with a minimum of 50 employees (e.g., industrial areas, mining areas, healthcare institutions, airports, nuclear power plants, and military installations) located outside urban areas [51]. Thus, not all workplace neighborhoods lie within residential areas, and we had missing data for the workplace neighborhood income, low education and unemployment. For sensitivity analyses, we imputed missing values with mean values from the year in question before creating the summary SES score to test if gaining statistical power changed our results. Finally, we ran analyses using a larger, 1,000 m buffer for the neighborhood exposures.

Lowest SES quartile was used as a reference. Effect estimates are expressed as odds ratios (OR) with 95% confidence interval (CI). All analyses were performed using SAS 9.4.

## Results

A total of 12,932 participants responded to a minimum of two surveys between 2012 and 2018 and had informaiton on all covariates and exposure. Descriptive statistics of the study population are provided in Table 1. The participants had a mean age of 50 years (range 20–75) at the time of their first response and slightly more than half (57%) were women. Over 82% of the participants were working full time and a majority (48%) held intermediate occupational positions. Fourteen percent of the participants both lived and worked in a neighborhood with the highest SES, while 3.6% lived in the highest but worked in the lowest SES neighborhood. The unemployment rate was very low in both home and work neighborhoods (Table 1). There were 3,224 individuals who had a change in their home neighborhood SES between two survey waves: 2,731 had one, 464 had two, and 29 had three changes. Work neighborhood SES was changed among 3,684 individuals: 2,935 had one, 696 had two, and 53 had three changes.

**Table 1.** Characteristics of the study participants eligible for the analysis in the first and last measurement point

Variables	First	Last
	*N* = 12,932	*N* = 12932
**Covariates**	%	%
Sex		
Women	58	58
Presence of children	28	21
Marital status		
Cohabiting	80	79
Occupational position		
Low	28	27
Intermediate	48	47
High	24	25
Self-employed	0.8	0.8
Chronic disease	41	45
Depressive symptoms	14	15
Job strain	20	20
**Outcomes**		
Obese	19	22
Physically inactive	19	19
Sedentary behavior	15	13
Smoking	10	9
Risky alcohol consumption	7	7
Disturbed sleep	19	20
**Exposures**	Mean (*SD*)	
Home neighborhood SES		
Mean income (SEK)		
First quartile	214,080 (31,350)	235,850 (34,200)
Second quartile	251,660 (25,840)	277,740 (26,390)
Third quartile	279,060 (28,570)	306,830 (30,700)
Fourth quartile	336,920 (59,340)	373,070 (67,700)
Low education (%)		
First quartile	0.78 (0.08)	0.76 (0.08)
Second quartile	0.69 (0.07)	0.67 (0.07)
Third quartile	0.59 (0.08)	0.56 (0.08)
Fourth quartile	0.43 (0.09)	0.41 (0.09)
Unemployment (%)		
First quartile	0.03 (0.03)	0.03 (0.03)
Second quartile	0.02 (0.02)	0.02 (0.02)
Third quartile	0.02 (0.01)	0.02 (0.01)
Fourth quartile	0.02 (0.02)	0.01 (0.02)
Work neighborhood SES		
Mean income (SEK)		
First quartile	195,280 (34,210)	210,180 (37,760)
Second quartile	236,590 (28,390)	260,250 (28,170)
Third quartile	265,790 (34,610)	292,140 (31,250)
Fourth quartile	338,080 (63,520)	370,110 (67,730)
Low education (%)		
First quartile	0.72 (0.10)	0.71 (0.09)
Second quartile	0.67 (0.11)	0.65 (0.10)
Third quartile	0.55 (0.11)	0.53 (0.09)
Fourth quartile	0.38 (0.08)	0.36 (0.07)
Unemployment (%)		
First quartile	0.05 (0.02)	0.05 (0.04)
Second quartile	0.03 (0.01)	0.03 (0.01)
Third quartile	0.02 (0.01)	0.02 (0.01)
Fourth quartile	0.02 (0.01)	0.02 (0.01)

**Fig. 1.**
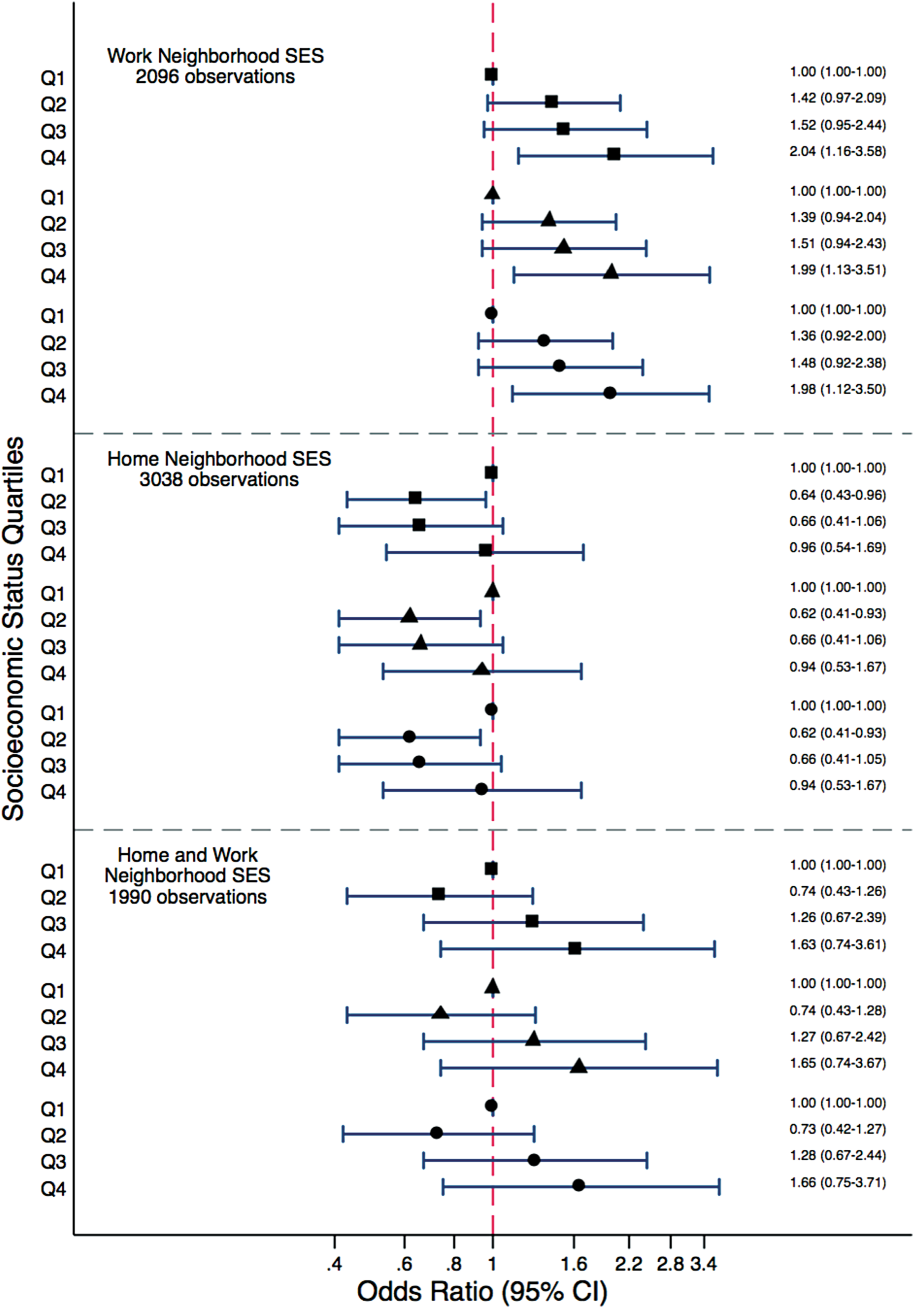
Associations between work, home, and time weighted home and work neighborhood socioeconomic status (SES) and risky alcohol consumption among gainfully employed individuals. Square markers indicate results from Model 1 adjusted for age, occupational position, marital status, and presence of children under 12; triangle markers indicate results from Model 2 additionally adjusted for chronic disease, and depressive symptoms; and circle markers from Model 3 subsequently adjusted for job strain. Q1, quartile 1; Q2, quartile 2; Q3, quartile 3; Q4, quartile 4.

We observed that the SES of the workplace neighborhood was associated with risky alcohol consumption (Fig. 1). When the participants worked in a high SES neighborhood they were more likely to engage in risky alcohol consumption compared to the time when their workplace was located in the low SES neighborhood (fully adjusted OR 1.98, 95% CI: 1.12, 3.5). Although the SES of the home neighborhood had a significant negative association with risky alcohol consumption in the second-lowest SES quartile, estimates approached one as the participants moved to the higher SES home neighborhood (Fig. 1). For the time-weighted home and work neighborhood SES, effect estimates indicated that higher neighborhood SES was associated with an increased risk of risky alcohol consumption, however, associations were not statistically significant (Fig. 1). The associations between work and home neighborhood SES and risky alcohol consumption were not modified by sex (*p*-values for interaction ranged between .3 and .9).

Regarding the other outcomes (Table 2), we observed an indication that participants might be at an increased risk of obesity when their workplace was located in the highest SES neighborhood compared to a time when their workplace was located in a neighborhood with the lowest SES (fully adjusted OR 1.71, 95% CI 1.02 to 2.87). However, no associations were observed between home and time-weighted average home and work neighborhood SES and obesity (Tables 3 and 4).

**Table 2.** Associations between work neighborhood socioeconomic status and health-related behaviors among gainfully employed individuals

Outcomes	Work neighborhood socioeconomic status			
	First (ref) quartile	Second quartile OR (95% CI)	Third quartile OR (95% CI)	Fourth quartile OR (95% CI)
**Obesity (2,646)**				
Model 1^a^	1	1.33 (0.94–1.88)	1.04 (0.68–1.59)	1.65 (0.99–2.77)
Model 2^b^	1	1.31 (0.92–1.86)	1.02 (0.67–1.56)	1.64 (0.98–2.74)
Model 3^c^	1	1.33 (0.93–1.89)	1.05 (0.68–1.61)	1.71 (1.02–2.87)
**Physical inactivity (5,374)**				
Model 1^a^	1	1.04 (0.83–1.31)	1.04 (0.80–1.36)	1.09 (0.80–1.48)
Model 2^b^	1	1.03 (0.82–1.30)	1.04 (0.80–1.36)	1.09 (0.80–1.48)
Model 3^c^	1	1.03 (0.82–1.30)	1.04 (0.80–1.36)	1.09 (0.80–1.48)
**Smoking (1,362)**				
Model 1^a^	1	1.20 (0.72–2.00)	0.63 (0.33–1.19)	1.28 (0.62–2.65)
Model 2^b^	1	1.20 (0.72–2.00)	0.63 (0.33–1.21)	1.29 (0.62–2.68)
Model 3^c^	1	1.20 (0.72–2.01)	0.63 (0.33–1.21)	1.29 (0.62–2.68)
**Sedentariness (4,694)**				
Model 1^a^	1	0.91 (0.71–1.17)	0.84 (0.62–1.15)	0.79 (0.55–1.12)
Model 2^b^	1	0.90 (0.70–1.16)	0.85 (0.62–1.17)	0.80 (0.56–1.14)
Model 3^c^	1	0.91 (0.70–1.17)	0.86 (0.63–1.18)	0.81 (0.57–1.16)
**Disturbed sleep (8,120)**				
Model 1^a^	1	0.94 (0.77–1.15)	0.96 (0.76–1.21)	1.02 (0.78–1.35)
Model 2^b^	1	0.92 (0.76–1.13)	0.97 (0.76–1.23)	1.06 (0.80–1.40)
Model 3^c^	1	0.92 (0.75–1.12)	0.97 (0.76–1.23)	1.06 (0.78–1.40)

^a^Adjusted for age, occupational position, marital status, and presence of children under 12.

^b^Adjusted for age, occupational position, marital status, presence of children under 12, chronic disease, and depressive symptoms.

^c^Adjusted for age, occupational position, marital status, presence of children under 12, chronic disease, depressive symptoms, and job strain.

**Table 3.** Associations between home neighborhood socioeconomic status and health-related behaviors among gainfully employed individuals

Outcomes	Home neighborhood socioeconomic status			
	First (ref) quartile	Second quartile OR (95% CI)	Third quartile OR (95% CI)	Fourth quartile OR (95% CI)
**Obesity (4,017)**				
Model 1^a^	1	0.76 (0.53–1.10)	0.78 (0.49–1.23)	0.90 (0.52–1.57)
Model 2^b^	1	0.76 (0.53–1.10)	0.77 (0.49–1.22)	0.89 (0.51–1.56)
Model 3^c^	1	0.75 (0.52–1.08)	0.76 (0.48–1.19)	0.88 (0.50–1.53)
**Physical inactivity (7,475)**				
Model 1^a^	1	0.99 (0.77–1.27)	0.90 (0.67–1.22)	0.72 (0.51–1.03)
Model 2^b^	1	0.99 (0.77–1.27)	0.90 (0.67–1.22)	0.73 (0.51–1.03)
Model 3^c^	1	0.99 (0.77–1.27)	0.90 (0.67–1.22)	0.73 (0.51–1.04)
**Smoking (1,965)**				
Model 1^a^	1	1.04 (0.65–1.66)	0.79 (0.42–1.47)	1.49 (0.67–3.31)
Model 2^b^	1	1.05 (0.65–1.68)	0.79 (0.42–1.47)	1.48 (0.67–3.29)
Model 3^c^	1	1.05 (0.65–1.68)	0.79 (0.42–1.47)	1.48 (0.67–3.29)
**Sedentariness (6,660)**				
Model 1^a^	1	1.04 (0.78–1.39)	0.82 (0.58–1.18)	0.94 (0.63–1.41)
Model 2^b^	1	1.06 (0.79–1.41)	0.83 (0.58–1.19)	0.95 (0.63–1.42)
Model 3^c^	1	1.06 (0.79–1.42)	0.82 (0.58–1.18)	0.96 (0.64–1.44)
**Disturbed sleep (11,162)**				
Model 1^a^	1	0.97 (0.78–1.20)	1.00 (0.77–1.30)	0.96 (0.70–1.31)
Model 2^b^	1	0.96 (0.78–1.20)	1.00 (0.76–1.30)	0.94 (0.68–1.30)
Model 3^c^	1	0.96 (0.78–1.20)	1.00 (0.76–1.30)	0.94 (0.68–1.30)

^a^Adjusted for age, occupational position, marital status, and presence of children under 12.

^b^Adjusted for age, occupational position, marital status, presence of children under 12, chronic disease, and depressive symptoms.

^c^Adjusted for age, occupational position, marital status, presence of children under 12, chronic disease, depressive symptoms, and job strain.

**Table 4.** Associations between weighted average of home and work neighborhood socioeconomic status and health-related behaviors among gainfully employed individuals

Outcomes	Home and work neighborhood socioeconomic status (quartile)			
	First (ref) quartile	Second quartile OR (95% CI)	Third quartile OR (95% CI)	Fourth quartile OR (95% CI)
**Obesity (2,487)**				
Model 1^a^	1	1.12 (0.70–1.80)	0.99 (0.54–1.79)	0.87 (0.42–1.79)
Model 2^b^	1	1.14 (0.71–1.82)	0.97 (0.53–1.76)	0.85 (0.41–1.76)
Model 3^c^	1	1.12 (0.70–1.80)	0.97 (0.53–1.76)	0.84 (0.41–1.74)
**Physical inactivity (5307)**				
Model 1^a^	1	1.05 (0.80–1.39)	0.91 (0.64–1.28)	1.08 (0.70–1.65)
Model 2^b^	1	1.04 (0.79–1.37)	0.90 (0.64–1.27)	1.07 (0.70–1.64)
Model 3^c^	1	1.04 (0.79–1.38)	0.90 (0.64–1.27)	1.07 (0.70–1.64)
**Smoking (1,264)**				
Model 1^a^	1	0.69 (0.32–1.47)	0.79 (0.29–2.12)	1.64 (0.52–5.18)
Model 2^b^	1	0.69 (0.32–1.47)	0.79 (0.29–2.12)	1.62 (0.51–5.13)
Model 3^c^	1	0.70 (0.32–1.48)	0.79 (0.29–2.13)	1.63 (0.52–5.14)
**Sedentariness (4,370)**				
Model 1^a^	1	0.91 (0.62–1.34)	0.95 (0.59–1.55)	1.05 (0.59–1.86)
Model 2^b^	1	0.89 (0.61–1.31)	0.95 (0.59–1.55)	1.03 (0.58–1.83)
Model 3^c^	1	0.90 (0.61–1.32)	0.96 (0.59–1.55)	1.04 (0.59–1.85)
**Disturbed sleep (7,591)**				
Model 1^a^	1	1.02 (0.78–1.33)	1.06 (0.76–1.48)	1.20 (0.73–1.65)
Model 2^b^	1	1.01 (0.77–1.33)	1.07 (0.76–1.49)	1.09 (0.72–1.65)
Model 3^c^	1	1.02 (0.78–1.34)	1.08 (0.77–1.51)	1.09 (0.72–1.66)

^a^Adjusted for age, occupational position, marital status, and presence of children under 12.

^b^Adjusted for age, occupational position, marital status, presence of children under 12, chronic disease, and depressive symptoms.

^c^Adjusted for age, occupational position, marital status, presence of children under 12, chronic disease, depressive symptoms, and work strain.

We observed no associations for the three neighborhood SES variables with physical inactivity, smoking, sedentariness, or disturbed sleep (Tables 2–4).

### Sensitivity Analyses

When associations between home neighborhood SES and risky alcohol consumption were adjusted for work neighborhood SES, estimates remained similar but confidence intervals became wider as the number of observations dropped (3,038 vs. 2,096) due to missing data on work neighborhood SES (Supplement Table 1). However, associations between work neighborhood SES and risky alcohol consumption remained robust after adjusting for home neighborhood SES. A similar pattern was observed for work neighborhood SES and obesity. After imputing zero values with mean values in the workplace neighborhood SES determinants, the effect estimates for risky alcohol consumption were attenuated but remained significant for the highest SES category (Supplement Table 2). For obesity, associations in the highest SES quartile became weaker and nonsignificant.

Results for 1,000 m buffer demonstrated a higher likelihood of risky alcohol use when participants lived in a high SES neighborhood compared to the time when they lived in a low SES neighborhood, however, confidence intervals were very wide and nonsignificant (Supplement Table 3). The risk estimates for work neighborhood SES and risky alcohol consumption increased as the SES improved, but were statistically nonsignificant. For obesity, work neighborhood SES demonstrated similar patterns as the 500 m buffer but of a weaker magnitude (Supplement Table 3).

## Discussion

In this within-individual study, we observed associations between SES of work neighborhood and an increased risk of risky alcohol consumption in gainfully employed individuals. For home neighborhood SES, the effect estimates were mainly in the opposite direction though nonsignificant for the highest SES quartiles. There was also an indication that while working in the highest SES neighborhoods individuals might more likely be obese compared to the time when they were working in the lowest SES neighborhood. No associations were observed for the other outcomes.

In our study, we observed no consistent associations between home neighborhood SES and health-related behaviors. Although living in the second lowest versus lowest home neighborhood SES quartile was associated with a decreased likelihood of risky alcohol consumption, the association was not consistent through the SES quartiles. Large systematic reviews comprising mainly of cross-sectional studies, have reported limited and conflicting support for the association between area-level disadvantage and increased alcohol use with some findings supporting the hypothesis while others pointing in the opposite direction [28, 52, 53]. Longitudinal studies with varying methodological approaches have reported associations of increased neighborhood poverty and neighborhood SES with binge drinking [54] and decrease in weekly alcohol consumption [55], respectively. Conversely, high urbanization and high SES have also been associated with increased risky alcohol consumption [30]. The inconsistent home neighborhood SES results might stem from heterogeneity among studies in terms of analytical methods applied [29], classification of alcohol intake [56], differences in socioeconomic gradients and neighborhood structures in different countries, as well as differences in study populations [29, 56]. We used a within-individual approach, that is, fixed-effect method, which is effective in reducing the confounding effects of unmeasured time-invariant causes. However, its focus on within-individual variance reduces its statistical power [29] and might thus provide weaker effect estimates [30]. Moreover, it is likely that we might not have captured all hazardous drinkers since our study participants were gainfully employed which already puts them in a higher stratum in the socioeconomic gradient.

Interestingly, our results for work neighborhood SES and alcohol use and obesity were in a different direction than those reported in previous studies regarding home neighborhood SES. This was also contrary to our hypothesis, though the hypothesis was based mainly on studies using home neighborhood SES. To the best of our knowledge, this is the first within-individual study to investigate the relationship between work neighborhood SES and behavior-related health. Previously, only two studies have considered work neighborhood SES and both were cross-sectional. One study focused on school teachers in Finland and reported an increased risk of heavy alcohol consumption in teachers working in poor vs. wealthy neighborhoods [26]. Our study sample represents working Swedish population from all occupations and these workplaces might have more variability in work and social cultures. It could be that social comparisons at workplace might be underlying the observed associations of this study. As a post-hoc analysis, we checked if individual SES had an interaction with work neighborhood SES in the model for risky alcohol consumption. Such interaction did not exist (*p*-value = .93) and thus social comparison effects, that is, employees with lower individual SES would drink more if they see a richer environment and people during the day, is unlikely to explain our finding. However, likely explanation could be that in some occupations after-work drinks with colleagues to decompress a long day or a week, celebrating wins or new clients, welcomes and farewells to a colleague, and weekend parties at pubs may be more common and considered important to climb the corporate ladder. Availability of alcohol may be another factor as higher SES workplace neighborhoods are often concentrated with on-premise and off-premise alcohol outlets making access to alcohol for people working in these neighborhoods easier. Overall, it is likely that competitive, stressful or nonrewarding work in combination with social norms and values that support drinking [57] along with easy access to alcohol can lead to risky drinking. The second prior study investigated BMI in relation to workplace neighborhood SES in a working population of over 50 years of age and did not report significant associations [24]. In our study sample, including participants from all working-age groups, higher workplace neighborhood SES indicated some association with obesity. However, the association was not robust to different testing, so further studies are needed to confirm if such association exists. Importantly, future studies should consider associations between workplace neighborhood SES and health behaviors in urban areas only as we had no data to perform such restricted analyses.

This study has several strengths but also limitations. The major strength of our study is the within-individual study design with repeated measurements. This allowed us to examine if changes in neighborhood SES were associated with changes in health-related behaviors that strengthen causal inference. While the fixed effects’ focus on within-individual variation might cause a lack of precision, it is likely to provide less confounded estimates as it controls for many unmeasured time-invariant factors by design [29]. However, we cannot exclude potential confounding from unmeasured time-varying confounding. Another strength of our study is the use of a population-based sample. Previous studies that examined both home and non-home neighborhoods were generally confined to study populations from one geographical area whereas our study population is more geographically representative of the working Swedish population. This, however, limits the generalizability of our results regarding home neighborhoods to unemployed, student or retired populations. It is also likely that we did not capture all hazardous drinkers if they are more prevalent among the non-working populations.

We had data on the number of hours participants spent on workplace and commuting between home and workplace which enabled us to estimate time-weighted average of time spent at each neighborhood. We had some missing data for workplace neighborhoods that did not have residential buildings within 500 m radius around them. However, when missing data were imputed with mean values of the workplace neighborhood SES our results persisted. Moreover, for all neighborhood SES variables we used reliable routinely collected register-based statistics from Statistics Sweden. This information along with home and workplace addresses was available for end of each year. As the surveys were conducted in the spring we used exposure data from the end of the previous year. This mismatch and the lack of information on all address changes among the study participants might have caused some exposure misclassification. All our outcome measures were self-reported which might introduce social desirability bias [58], but the within-individual design reduces this type of bias as participants’ response styles are likely to be similarly biased from one survey to another.

## Conclusions

Workplace neighborhood SES was associated with a higher risk of risky alcohol consumption. Findings of our study suggest that the consideration of work neighborhood SES along with home neighborhood SES might enhance our understanding of how environments where individuals spend majority of their waking time influence their health-related behaviors. Also, in order to get more reliable estimates regarding the associations between neighborhood characteristics and health, more longitudinal within-individual comparisons are needed.

## Supplementary Material

kaaa116_suppl_Supplemental_Tables
