# Supplementary material for: Home and Workplace Neighborhood Socioeconomic Status and Behavior-related Health: A Within-individual Analysis
Source: Ann Behav Med. 2021 Feb 13;55(8):779–90. doi: 10.1093/abm/kaaa116 (PMC8311784; doi:10.1093/abm/kaaa116)
Supplement: kaaa116_suppl_Supplemental_Tables [file kaaa116_suppl_supplemental_tables.docx]

Supplemental Table 1: Associations for home and work, and weighted home and work neighborhood socioeconomic status and excessive alcohol use and obesity in all models with additional adjustment home and workplace neighborhood in respective exposures

| Outcomes (observations) | Home neighborhood socioeconomic status adjusted for work neighborhood socioeconomic status | | | |
| --- | --- | --- | --- | --- |
|  | 1^st^ (ref) quartile OR (95% CI) | 2^nd^ quartile  OR (95% CI) | 3^rd^ quartile  OR (95% CI) | 4^th^ quartile  OR (95% CI) |
| **Risky alcohol consumption** **(2096)** |  |  |  |  |
| Model 1^*^ | 1 | 0.65 (0.37-1.15) | 0.74 (0.40-1.37) | 0.99(0.49-2.00) |
| Model 2^#^ | 1 | 0.63 (0.36-1.11) | 0.73 (0.39-1.36) | 0.98 (0.48-2.00) |
| Model 3^¤^ | 1 | 0.64 (0.36-1.12) | 0.72 (0.39-1.36) | 0.97 (0.47-1.99) |
| **Obesity (2646)** |  |  |  |  |
| Model 1^*^ | 1 | 0.95 (0.57-1.58) | 0.92 (0.49-1.71 | 1.04 (0.51-2.12) |
| Model 2^#^ | 1 | 0.94 (0.57-1.56) | 0.92 (0.50-1.71) | 1.02 (0.50-2.09) |
| Model 3^¤^ | 1 | 0.93 (0.56-1.55) | 0.88 (0.47-1.64) | 0.99 (0.48-2.02) |
|  | **work neighborhood socioeconomic status adjusted for home neighborhood socioeconomic status** | | | |
| **Risky alcohol consumption** **(2096)** |  |  |  |  |
| Model 1^*^ | 1 | 1.41 (0.96-2.07) | 1.51 (0.94-2.43) | 2.04 (1.16-3.60) |
| Model 2^#^ | 1 | 1.37 (0.93-2.02) | 1.50 (0.93-2.41) | 2.00 (1.13-3.52) |
| Model 3^¤^ | 1 | 1.35 (0.91-1.98) | 1.46 (0.91-2.36) | 1.98 (1.12-3.50) |
| **Obesity** **(2646)** |  |  |  |  |
| Model 1^*^ | 1 | 1.33 (0.94-1.88) | 1.04 (0.68-1.59) | 1.65 (0.98-2.76) |
| Model 2^#^ | 1 | 1.31 (0.92-1.86) | 1.02 (0.66-1.56) | 1.63 (0.98-2.74) |
| Model 3^¤^ | 1 | 1.33 (0.93-1.88) | 1.05 (0.68-1.61) | 1.70 (1.01-2.86) |

^*^Adjusted for age, occupational position, marital status and presence of children under 12

^#^Adjusted for age, occupational position, marital status, presence of children under 12, chronic disease, and depressive symptoms

^¤^Adjusted for age, occupational position, marital status, presence of children under 12, chronic disease, depressive symptoms, and work strain

Supplemental Table 2: Associations for work neighborhood socioeconomic status and excessive alcohol use and obesity: sensitivity analysis using neighborhood SES index where missing values were replaced with mean values.

| Outcomes (observations) | Work neighborhood socioeconomic status | | | |
| --- | --- | --- | --- | --- |
|  | 1^st^ (ref) quartile OR (95% CI) | 2^nd^ quartile  OR (95% CI) | 3^rd^ quartile  OR (95% CI) | 4^th^ quartile  OR (95% CI) |
| **Risky alcohol consumption** **(2745)** |  |  |  |  |
| Model 1^*^ | 1 | 1.15 (0.83-1.57) | 1.10 (0.76-1.59) | 1.72 (1.10-2.69) |
| Model 2^#^ | 1 | 1.14 (0.83-1.56) | 1.12 (0.76-1.61) | 1.71 (1.09-2.67) |
| Model 3^¤^ | 1 | 1.13 (0.82-1.56) | 1.11 (0.76-1.61) | 1.72 (1.10-2.69) |
| **Obesity** **(3630)** |  |  |  |  |
| Model 1^*^ | 1 | 1.15 (0.87-1.5) | 0.97 (0.71-1.34) | 1.10 (0.73-1.64) |
| Model 2^#^ | 1 | 1.15 (0.88-1.52) | 0.98 (0.71-1.35) | 1.12 (0.75-1.68) |
| Model 3^¤^ | 1 | 1.15 (0.87-1.51) | 0.99 (0.71-1.36) | 1.14 (0.76-1.70) |

^*^Adjusted for age, occupational position, marital status and presence of children under 12

^#^Adjusted for age, occupational position, marital status, presence of children under 12, chronic disease, and depressive symptoms

^¤^Adjusted for age, occupational position, marital status, presence of children under 12, chronic disease, depressive symptoms, and work strain

Supplemental Table 3: Associations for home, work, and weighted home and work neighborhood socioeconomic status and excessive alcohol use and obesity in 1000-meter radius

| Outcomes (observations) | Home neighborhood socioeconomic status | | | |
| --- | --- | --- | --- | --- |
|  | 1^st^ (ref) quartile OR (95% CI) | 2^nd^ quartile  OR (95% CI) | 3^rd^ quartile  OR (95% CI) | 4^th^ quartile  OR (95% CI) |
| **Excessive alcohol use** **(3038)** |  |  |  |  |
| Model 1^*^ | 1 | 1.10 (0.71-1.71) | 1.06 (0.64-1.77) | 1.16 (0.63-2.14) |
| Model 2^#^ | 1 | 1.08 (0.70-1.69) | 1.04 (0.62-1.74) | 1.13 (0.61-2.09) |
| Model 3^¤^ | 1 | 1.09 (0.70-1.70) | 1.05 (0.63-1.76) | 1.14 (0.62-2.12) |
| **Obesity** **(4071)** |  |  |  |  |
| Model 1^*^ | 1 | 0.98 (0.67-1.42) | 1.01 (0.64-1.61) | 0.751 (0.41-1.36) |
| Model 2^#^ | 1 | 0.98 (0.68-1.43) | 1.01 (0.63-1.59) | 0.742 (0.41-1.35) |
| Model 3^¤^ | 1 | 0.98 (0.67-1.42) | 0.98 (0.62-1.55) | 0.724 (0.40-1.31) |
|  | **Work neighborhood socioeconomic status** | | | |
| **Excessive alcohol use** **(2096)** |  |  |  |  |
| Model 1^*^ | 1 | 0.71 (0.47-1.08) | 0.789 (0.476-1.30) | 1.259 (0.711-2.230) |
| Model 2^#^ | 1 | 0.71 (0.47-1.08) | 0.831 (0.502-1.37) | 1.242 (0.701-2.201) |
| Model 3^¤^ | 1 | 0.71 (0.47-1.08) | 0.816 (0.492-1.35) | 1.238 (0.697-2.20) |
| **Obesity** **(2646)** |  |  |  |  |
| Model 1^*^ | 1 | 1.39 (0.97-1.98) | 1.074 (0.69-1.67) | 1.32 (0.77-2.28) |
| Model 2^#^ | 1 | 1.39 (0.97-1.98) | 1.079 (0.69-1.68) | 1.33 (0.77-2.30) |
| Model 3^¤^ | 1 | 1.40 (0.98-2.01) | 1.12 (0.72-1.75) | 1.400 (0.81-2.43) |
|  | **Home and work neighborhood socioeconomic status** | | | |
| **Excessive alcohol use** **(2096)** |  |  |  |  |
| Model 1^*^ | 1 | 0.65 (0.383-1.10) | 0.68 (0.36-1.28) | 1.01 (0.46-2.19) |
| Model 2^#^ | 1 | 0.68 (0.400-1.16) | 0.70 (0.37-1.34) | 1.06 (0.48-2.32) |
| Model 3^¤^ | 1 | 0.67 (0.397-1.15) | 0.68 (0.36-1.30) | 1.03 (0.47-2.25) |
| **Obesity** **(2645)** |  |  |  |  |
| Model 1^*^ | 1 | 0.95 (0.62-1.45) | 0.81 (0.47-1.37) | 1.03 (0.51-2.08) |
| Model 2^#^ | 1 | 0.94 (0.62-1.44) | 0.79 (0.46-1.35) | 1.02 (0.50-2.08) |
| Model 3^¤^ | 1 | 0.93 (0.61-1.43) | 0.80 (0.47-1.37) | 1.04 (0.51-2.12) |

^*^Adjusted for age, occupational position, marital status and presence of children under 12

^#^Adjusted for age, occupational position, marital status, presence of children under 12, chronic disease, and depressive symptoms

^¤^Adjusted for age, occupational position, marital status, presence of children under 12, chronic disease, depressive symptoms, and work strain
